# Updating search strategies for systematic reviews using EndNote

**DOI:** 10.5195/jmla.2017.183

**Published:** 2017-07-01

**Authors:** Wichor Bramer, Paul Bain

Performing, writing, and publishing a systematic review take a long time. In a cohort of journal-published systematic reviews, Cochrane reviews, and health technology assessment reports, the median time lag between the stated last search date and publication was 61 weeks (interquartile range, 33–87 weeks) [1]. In the same cohort of reviews, 7% were out of date at the time of publication [2]. More recently, an examination of 182 systematic reviews performed at Erasmus Medical Centre showed that the median time between the first search and the appearance of the resulting review in PubMed was 89 weeks (interquartile range, 63–126 weeks).

To maximize the currency of a review, an update of the search is recommended before submission for publication. The Methodological Expectations of Cochrane Intervention Reviews (MECIR) standards requires: “Rerun or update searches for all relevant databases within 12 months before publication” [3]. Many handbooks and guidelines for performing systematic reviews state that search strategies should be updated regularly to keep track of newly added references on the topic [4–6].

Recent guidance from an international panel of authors, editors, clinicians, statisticians, information specialists, other methodologists, and guideline developers considered various aspects of updating reviews, including efficient searching. Such efficiencies included refinements based on the yield of the original search and incorporation of technological advances in searching. Garner et al. provided practical guidance on refining the original search in their appendix 2 [7].

The Cochrane handbook mentions in chapter 3.4.2.1 (“Re-executing the search”) using the last date of the original search as the beginning date for the update, which is common practice, but chapter 6.4.12 (“Updating searches”) does not describe a clear method [8]. The date that the record became accessible through searching, rather than the publication date, is the relevant field for updating. The user can choose from the thesaurus date (i.e., the date that the thesaurus terms were added), the date of the last metadata change, or the date of entry into the database. For example, the National Library of Medicine recommends using the Create date (CRDT in PubMed) field for its databases [7]. The MeSH date field (MHDA in PubMed), which is the date the Medical Subject Headings (MeSH) terms were added to the record, also has some advantages. However, some interfaces, such as Web of Science, do not provide record dates that could guide updating. In such cases, searchers can use publication date and a safe overlapping period, resulting in extensive duplication with records retrieved in the original search.

Complicating matters further, the search may have been modified since the last search date. For instance, new words may have been added to the original search strategy, based on relevant terms found in studies included in the original review. These novel terms need to be searched in all the databases that were queried in the original search from the original starting date, thus requiring even more complicated search structures and date ranges. Hence, to many authors, updating a search can seem to be a complicated and uncertain task. It need not be.

The authors have developed a method for updating existing reviews that uses EndNote reference management software [9]. The technique uses two EndNote files: one containing the current results as they are downloaded from the complete set of databases, as if it were a first search; and one with the results of the previous or original search. By subtracting records found in the original search from the current results through EndNote’s customizable de-duplication feature, only records that were not screened in the original search will remain in the library. Another group has previously alluded to a similar process: “download all references from the update search and directly de-duplicate them with the references from the initial search (e.g., using Endnote)” [10].

Here, we describe the process in step-by-step detail. The steps will be identical in any recent version of EndNote. The method was first developed with the Microsoft Windows version of EndNote X3 and has been fairly consistent until the current version, X8. The same steps are also applicable for Macintosh editions by replacing the standard Windows controls with the corresponding Macintosh controls: for instance, command-A instead of control-A or command-click instead of control-click.

## A NEW METHOD FOR UPDATING EXISTING SEARCHES

The initial search requires no extra action. We do recommend thorough de-duplication in EndNote by the process described in an earlier article by Bramer et al. [11]. This earlier article also describes how substantial differences in the way single articles are represented in various databases can be resolved. It describes how page numbers from MEDLINE and the Cochrane Library can be completed, turning abbreviated pagination (e.g., 1008–12) into full format pagination (e.g., 1008–1012) as is used in other databases. The earlier article also recommends importing full journal titles instead of abbreviations. If these steps are followed, the method described in this article will be easier to follow. The following description will still be effective if the libraries were de-duplicated using other sequences in EndNote but will require additional care to ensure that novel articles are not eliminated.

The first steps in the updating process serve to create an EndNote file containing all results from the new or current search as they are retrieved on the new date. This search’s date should now be considered the last date searched, and the number of records retrieved from this search will be used in the published PRISMA flow chart. In the process, a compressed library (.enlx file) can serve as an archive and be used in subsequent updates to identify and remove previously screened records.

### Step 1: Rerun the search

1. If search terms are to be changed or added, make those changes to the search strategies.
2. Run the searches in all databases from the original starting date (rather than limiting from the previous search date) and import all results into EndNote.
3. De-duplicate the EndNote file (preferably as described by Bramer et al. [11]).
4. Create a compressed EndNote Library (.enlx file) of the complete search results, and store it for possible use in future updates. This set of results should be reported in the published review as the number of records reviewed by title and/or abstract. A method to review titles and abstracts in EndNote is published in a previous article by Bramer et al. [12].

Records from the original search have already been screened and need not be seen again. To remove these records from the current search results, the old records are added to the EndNote Library containing the records from the new search. By means of duplicate detection, matching pairs of records (one from the original search and the other from the new search) can be identified and removed, leaving only new records that have yet to be screened.

### Step 2: Copy the original search results in the new EndNote file

1. Open the EndNote library that contains all results found on the last search date before the current date. If this was an update of an earlier search, use the complete compressed library that was created at that time.
2. Press <Ctrl-A> to select all references. Right-click on one of the references and from the resulting menu, select Copy References to. Now, select the EndNote Library containing the results of the current search. This will move all the records in this library to the new library. Copied records will appear in a new group called Copied References. Using the Copy References to command instead of copying and pasting or importing records facilitates the identification of records from the previous search.

### Step 3: Identify and remove records retrieved by both the previous and new searches

1. Go to the EndNote file containing the old and new references.
2. Modify the default duplicate settings by going to Edit > Preferences > Duplicates. (The Preferences menu option can be found under the EndNote menu on the Mac.) Select the fields: Author, Year, Title, Secondary Title (Journal).
3. Select one random reference in the All References group to ensure that this group is active.
4. Go to References > Find Duplicates. Click on Cancel in the Find Duplicates dialog box to reveal the Duplicate References group.
5. Press <Ctrl-A> to select all references in the Duplicate References group. Press <Delete> to remove all references in the group, or drag the references to the trash.
6. Repeat step 3: 2–5 for the Author, Year, Title, Pages fields.

If the original search was executed in the recent past and used the same methods for import into EndNote, the Copied References group should be nearly empty after this step. If the original search was performed long before the update took place or the initial search had been performed by another searcher, possibly using different export methods and interfaces, there may be a large number of remaining unmatched references from the original search in the Copied References group. If so, follow two extra steps of the method described in Bramer et al. [11]. Step 3: 2–5 can be repeated with the Title, Volume, Pages fields and then with the Author, Volume, Pages fields; however, in these cases, references without page numbers should be assessed for duplicates, and true duplicates should be manually selected. To do this, after step 3: 4, click on the Pages column heading to sort by page number. References with page numbers can be deleted without manual assessment. If the Pages column heading is not visible in the reference table, go to Edit > Preferences > Display Fields, and select the Pages field in one of the columns.

### Step 4: Compare old and new records on similar titles or authors

In the next steps, the new results are compared with the original results on title. This requires quick manual assessment to prevent removing two new, independent search results that share the exact title.

**Figure 1:**
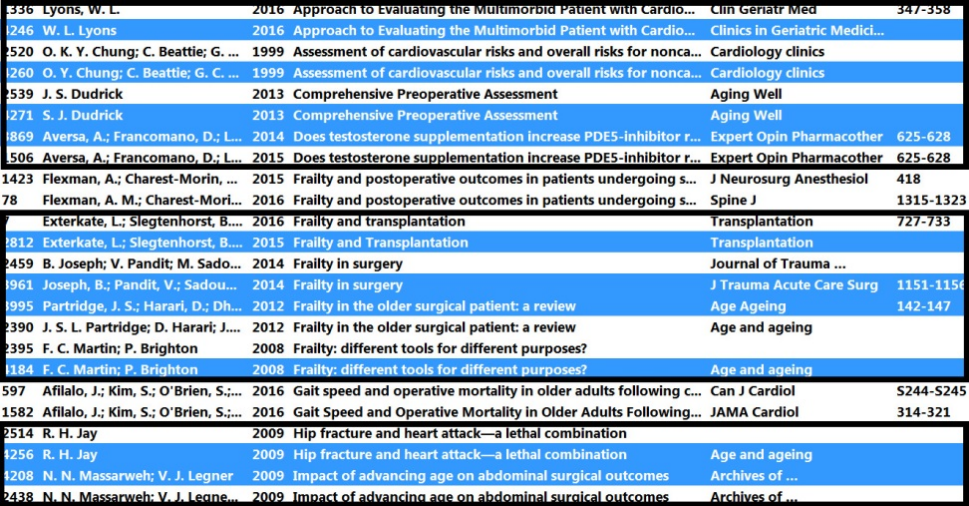
Recognition of pattern change in blue and white references, indicating a duplicate that should not be removed; the references in the black boxes are updates and should all be removed

1. Go to Edit > Preferences > Duplicates, and select only the Title field. (The Preferences menu option can be found under the EndNote menu on the Mac.)
2. Select one random reference in the All References group to ensure the group is active.
3. Go to References > Find Duplicates, and click Cancel in the next screen.
4. Go to the Copied References group, select one reference and press <Ctrl-A> to select all references from the previous search.
5. Go to the Duplicate References group to scan this set for duplicates. Click on the Title column heading to sort by title. True duplicates can be easily found by observing the pattern of blue and white lines. As long as white lines and blue lines are in a somewhat regular, one-by-one pattern, the pairs will consist of one reference from the old results and one reference from the new results. Occasionally, groups of two blue or two white lines might appear because the sorting is irregular. If, however, a group of more than two white lines is observed or two consecutive groups of two white references, this means that some of these references are not from the previous search results but are two current records with identical titles that represent different articles. These should not be selected for deletion. Figure 1 shows an example of such a pattern change. In this case, the references from authors Flexman and Afilalo should not be deleted, as they are not duplicates.
6. Select references in sequences with regular blue-white patterns by using <Ctrl-Click> to select the beginning of such a sequence and <Shift-Click> to end a selection. This might require some practice if the user is unfamiliar with this option.
7. After all regular sequences have been selected, press <Delete> to remove all duplicate references or drag the duplicate references to the trash.
8. Repeat step 4: 1–7 for the Author, Year field combination.
9. Go to the Copied References folder, and delete any references in that folder. These are the references that were found in the previous search but that were not found in the current search. They should not be reviewed again.

## CORRECT REPRESENTATION IN THE PRISMA FLOW CHART

A consequence of this method is that the reference numbers and totals required for the PRISMA flow chart might not match, as the sum of the novel and old records might not equal the contents of the new EndNote library. This discrepancy can be attributed to several normal database activities: updates to individual records (such as added volume and page numbers or a changed publication year after an article appeared in print), global changes to controlled vocabulary, recent addition of older material to the database, or the dropping out of high relevance ranking in Google Scholar. Additionally, records with publication dates that antedate the previous search will appear among the novel records. This can be the result of both changes to controlled vocabulary. Revising and adding to the search strategy will also result, appropriately, in retrieving novel records from before the date of the previous search as well. Hence, the most reliable method of correcting the numbers for publication in the PRISMA flowchart is backward correction.

Typically, the total number of unique records retrieved is reported both in the results section of a systematic review and in the PRISMA flow chart detailing the search and subsequent screening. For updated searches, the most appropriate number to report is the total number of records remaining in the updated search after removing duplicates. This number represents the minimum number of records screened for all searches. For dates of coverage, authors need only report the beginning date of coverage for each database and the date of the latest update, as the numbers of records are accurate for all searches at this point in time. The total number of full-text articles read will be the sum of the articles read in all the previous searches plus the number of articles read in current search. The number of articles excluded based on title abstract, for which no specific reason has to be given, is the total number of records remaining in the updated search minus the total number of full-text articles read. The numbers of records or articles excluded for the specific reasons in the full-text review stage can be summed over the previous and updated searches.

The records described in step 4: 9 are records that had been found during the original search but that were not found in the update search. This situation can occur if a search strategy during the update is narrowed compared to the original search strategy or if certain databases that had been searched initially were not searched during the update. If the search strategy has been narrowed, it is necessary to assure that the included references from the original search results are still retrieved by the current searches.

## DISCUSSION

We provide a method to simply subtract previously screened results from updated systematic review searches. By using this method, searchers spare themselves the time and effort required to reconcile update dates across platforms. Our method is described for use with EndNote bibliographic management software, although it might conceivably be adapted to other bibliographic managers. However, for optimal implementation of this method, such a program requires customizable settings for duplicate detection and the option to remove both duplicate references. We are unaware of a bibliographic manager that is as flexible as EndNote in this regard. Most software uses a standard de-duplication algorithm and is set to merge duplicate references instead of removing them.

There is no consistent, widely accepted method of updating searches. Our method suggests a simple standard for both carrying out and reporting updates that requires giving only the total number of records from the inception of the original search to the date of the last update, along with any revisions to the search itself. In reporting only these details, authors give an accurate representation of the state of the database and the response to the query on the date of the last search. The only information that is lost is the number of records retrieved and screened in previous searches that did not match records in the updated query. Rerunning and de-duplicating the updated search obviates the confusing task of choosing and reporting a date field for the beginning date of the new search (e.g., publication date, thesaurus date, creation date).
